# Self-Reported Nutritional Factors Are Associated with Weight Loss at 18 Months in a Self-Managed Commercial Program with Food Categorization System: Observational Study

**DOI:** 10.3390/nu13051733

**Published:** 2021-05-20

**Authors:** Ellen S. Mitchell, Qiuchen Yang, Annabell S. Ho, Heather Behr, Christine N. May, Laura DeLuca, Andreas Michaelides

**Affiliations:** 1 Academic Research, Noom, 229 W 28th St., New York, NY 10001, USA; qiuchen@noom.com (Q.Y.); annabell@noom.com (A.S.H.); hbehr@saybrook.edu (H.B.); christinem@noom.com (C.N.M.); ldeluca@mail.yu.edu (L.D.); andreas@noom.com (A.M.); 2 Department of Integrative Health, Saybrook University, 55 W Eureka Street, Pasadena, CA 91103, USA; 3 Ferkauf Graduate School of Psychology, Yeshiva University, 1165 Morris Park Avenue, Bronx, NY 10461, USA

**Keywords:** technologies, weight loss, obesity, energy density, nutrition knowledge, food choice

## Abstract

Little is known about nutritional factors during weight loss on digital commercial weight loss programs. We examined how nutritional factors relate to weight loss for individuals after 4 and 18 months on a mobile commercial program with a food categorization system based on energy density (Noom). This is a two-part (retrospective and cross-sectional) cohort study. Two time points were used for analysis: 4 months and 18 months. For 4-month analyses, current Noom users who met inclusion criteria (*n* = 9880) were split into 5% or more body weight loss and stable weight loss (0 ± 1%) groups. Individuals who fell into one of these groups were analyzed at 4 months (*n* = 3261). For 18-month analyses, individuals from 4-month analyses who were still on Noom 18 months later were invited to take a one-time survey (*n* = 803). At 18 months 148 participants were analyzed. Noom has a system categorizing foods as low-, medium-, and high-energy-dense. Measures were self-reported proportions of low-, medium-, and high-energy-dense foods, and self-reported nutritional factors (fruit and vegetable intake, dietary quality, nutrition knowledge, and food choice). Nutritional factors were derived from validated survey measures, and food choice from a novel validated computerized task in which participants chose a food they would want to eat right now. ANOVAs compared participants with 5% or more body weight loss and participants with stable weight (0 ± 1%) at 4 months on energy density proportions. Analyses at 18 months compared nutritional factors across participants with >10% (high weight loss), 5–10% (moderate weight loss), and less than 5% body weight loss (low weight loss), and then assessed associations between nutritional factors and weight loss. Individuals with greater weight loss reported consuming higher proportions of low-energy-dense foods and lower proportions of high-energy-dense foods than individuals with less weight loss at 4 months and 18 months (all ps < 0.02). Individuals with greater weight loss had higher fruit and vegetable intake (*p* = 0.03), dietary quality (*p* = 0.02), nutrition knowledge (*p* < 0.001), and healthier food choice (*p* = 0.003) at 18 months. Only nutrition knowledge and food choice were associated with weight loss at 18 months (B = −19.44, 95% CI: −33.19 to −5.69, *p* = 0.006; B = −5.49, 95% CI: −8.87 to −2.11, *p* = 0.002, respectively). Our results highlight the potential influence of nutrition knowledge and food choice in weight loss on a self-managed commercial program. We also found for the first time that in-the-moment inclination towards food even when just depicted is associated with long-term weight loss.

## 1. Introduction

Obesity is associated with amplified risk of health conditions such as cardiovascular disease and metabolic conditions [1,2,3]. Body weight loss of 5–10% can reduce the risk of these conditions [4]. It is estimated that many individuals use commercial programs, but there is little knowledge on how dietary quality or nutrition knowledge is impacted via these programs [5,6]. Most studies on nutritional factors in weight loss have taken place in non-commercial settings, such as clinical trials or free-living settings [7,8,9,10,11,12,13,14]. In digital commercial programs, however, individuals self-initiate and self-manage their participation with the setting and timeline of their choice. It is largely unknown how nutritional factors relate to weight loss on a self-managed commercial program. On one hand, previous studies found that individuals with varying degrees of self-management who maintained greater weight loss had healthier diets and more nutritional knowledge than individuals with less weight loss [15,16]. This suggests that nutritional factors such as better dietary quality or nutrition knowledge may be associated with greater weight loss on a self-managed commercial program. On the other hand, when self-managing their own weight loss, some individuals follow diets that are unsustainable and may restrict nutritional value long-term [15,16,17,18,19,20]. In addition, greater weight loss could be indicative of lower nutritional value [16,19]. It is possible that greater weight loss is associated with less optimal nutritional factors, or that associations between weight change and nutritional factors are not sustained long-term. These questions highlight the need to examine long-term nutritional factors in a self-managed commercial weight loss program to inform improving nutritional intake and knowledge for the large number of individuals who use these programs.

A number of studies suggest that energy density is associated with weight loss and healthy nutritional factors [21,22,23,24]. Energy density is the amount of energy in a food per weight (kcal/g) [24]. Individuals who eat a greater proportion of low-energy-dense foods consume fewer calories overall but report feeling just as full [25]. Along these lines, randomized controlled trials have found that individuals encouraged to eat low-energy-dense foods lose more weight and consume more foods with high micronutrient content like fruits and vegetables [21,22,26]. Low-energy-dense food intake is also associated with higher consumption of Vitamins A, C, and B-6 [10]. To our knowledge, no study has assessed the direct association between energy dense food choices and nutrition knowledge during weight loss. Since energy density and nutrition knowledge are each associated with better diet quality in general populations [10,27,28,29,30], there is a possibility that energy density could in turn be associated with nutrition knowledge during weight loss. At the same time, severely restricting diet to low energy density foods may not be sustainable long-term and lead to frustration [31]. Therefore, a flexible food system such as color coding that guides individuals towards low-energy-dense foods but allows moderate consumption of medium and high-density foods could be beneficial, particularly long-term [32]. 

In this study, we explored how nutritional factors are associated with weight loss in individuals on Noom, a self-managed commercial weight loss program with a food color categorization system based on energy density. One aim of the study was to understand how energy density relates to short-term and long-term weight loss. We hypothesized that users with more weight loss at 4 months and 18 months would eat lower energy dense diets than individuals with less weight loss or stable weight. The study’s primary aim was to examine how nutritional factors at 18 months are associated with weight loss. We hypothesized that fruit and vegetable intake, dietary quality, nutrition knowledge, and food choice would all be associated with weight loss at 18 months. Exploring the relation between weight loss and nutritional factors retrospectively can provide understanding of what is currently occurring for individuals on self-managed commercial programs and inform how to improve them to aid in short-term and long-term nutrition.

## 2. Materials and Methods

### 2.1. Study Design

A two-part observational cohort design was used to measure, to the extent possible, real-world use of Noom. To explore short-term energy density and weight loss, Noom users’ self-reported food and weight were extracted from the Noom database each week up to 4 months. To assess long-term nutritional factors, the same users were asked to complete an online survey 18 months after starting the program. Their self-reported 18-month weight and food intake data were extracted from the program database. All participants granted informed consent for their de-identified data to be used for retrospective research when they signed up for Noom; they were given the option to opt out. This study was self-funded and conducted by Noom-employed scientists in order to evaluate characteristics associated with weight loss and energy density. 

### 2.2. Program and Food Categorization System

Noom is a mobile commercial behavior change program that has led to clinically significant short-term and long-term weight reduction [33,34]. After voluntarily signing up for the program, users receive a curriculum; logging features for food, weight, and exercise; a 1:1 coach; and an online group that provides social support led by a group coach. To report food intake, users input the type of food, brand (if applicable), and serving size. They can enter a custom entry or choose from a database developed by Noom of thousands of foods available in the United States, including both branded and generic foods (i.e., banana). Calories for each food are available in the database or users can input the number of calories for a custom food [35]. Users report foods consumed at each meal separately. Users can choose from metric (g) or imperial (oz) serving sizes or common serving sizes (e.g., tablespoons, cups, slices). Users are encouraged, but not required, to log their food intake every day for each meal. Based on empirical findings and federal recommendations, Noom uses a food color categorization system to encourage greater consumption of low-energy-dense foods (green, <1 calorie/g or 0.4 calories/mL) over medium-energy-dense (yellow, 1–2.4 calories/g or 0.4–1 calorie/mL) and high-energy-dense (red, >2.4 calories/g or 1 calorie/mL) foods [36,37]. Whole grain foods (e.g., whole wheat/grain breads, brown rice) are moved down into the nearest lower-energy-dense category because low-energy-dense diets tend to have many whole grain foods [38], and to encourage consumption of a wider variety of healthy foods. Users are encouraged to consume a ratio of 30% green, 45% yellow, and 25% red foods; these proportions are incorporated in food logging and in daily goals set by users with help from coaches. When users log a food, they immediately see whether it is a green, yellow, or red food, as well as the proportion of green, yellow, and red foods they have consumed that day. Users are provided education on healthy eating and proper nutrition from MyPlate recommendations [39], in order to enable high diet quality long term. Coaches provide users with support and information on behavior change principles, such as how to maintain dietary goals.

### 2.3. Participants

4 months. Noom users who had independently signed up for the program and provided informed consent were eligible for the study. To be included in 4-month analyses, participants had to have signed up for the Noom Healthy Weight program in June 2019, be located in the United States, have a body mass index (BMI) of 25 or higher, and still be on the program at 4 months. There were 9880 participants meeting these criteria. Users were excluded if they were not between 18–85 years old; did not log a meal or weigh in at least once a week, which was necessary for modeling purposes; and/or did not provide full baseline criteria of age, height, gender, and initial weight, leaving 9261 participants. Weight loss was calculated by subtracting weight at 4 months from baseline weight (0 weeks). Two weight loss groups were created based on past studies: moderate weight loss (5% or more body weight loss), since 5% short-term weight loss is considered a clinically meaningful amount of moderate weight loss [40]; and stable weight (0 ± 1% body weight loss) to explore differences when weight is not lost. These groups were chosen to examine if energy density proportions explain variability between successful (i.e., clinically significant weight loss) and unsuccessful (i.e., stable weight) individuals. There were 3261 participants remaining after weight loss groups were established (stable: 374, moderate weight loss: 2887). Participants who did not fall into one of these groups (*n* = 6000) had more than 1% but less than 5% body weight loss at 4 months. 

18 months. Of these 3261 participants, those who were still on Noom at 18 months and therefore could provide weight and food log data (*n* = 803), were asked to complete an online survey via email. The survey was composed of validated questionnaires on fruit and vegetable intake, dietary quality, nutrition knowledge, and food choice. No nutritional or dietary factors were used to exclude participants. Out of 803 survey invitations, 245 completed the survey. Of these participants, 148 logged their weight sometime between 68–74 weeks, which was used to determine weight loss at 18 months. Weight measurements reported between weeks 68–74 were carried forward if missing at 18 months. Weight loss was calculated by the difference in weight from baseline to 18 months. Because weight outcomes at 18 months can vary more widely than those at 4 months, participants were split into three groups: high (>10% body weight, *n* = 71), moderate (5–10%, *n* = 35), and low weight loss (<5%, *n* = 42). This was based on evidence that 10% long-term weight loss is optimal for benefits and 5–10% weight loss provides moderate benefit [41].These groups cover a continuous range from weight gain to >10% weight loss.

### 2.4. Measures

4 months. Participants’ self-reported weekly weight and food intake from baseline to 4 months were extracted from the Noom database in April, 2020 to unobtrusively explore energy density proportions and weight loss. Energy density proportions represented the total number of calories for each food reported in each energy density (color) category as a function of the total number of calories consumed per week. Three proportions were calculated: low-energy-dense (calories for green foods/the total number of calories that week), medium-energy-dense (calories for yellow foods/total calories), and high-energy-dense (calories for red foods/total calories). Weekly proportions were used as dependent variables for linear mixed models, and proportions were averaged over 4 months as dependent variables for ANOVAs. 

18 months. Long-term nutritional factors were measured in December, 2020 as outcomes 18 months after starting the program. The following validated survey assessments were used.

Fruit and vegetable intake. Self-reported fruit and vegetable intake was measured with the fruit and vegetable screener from the Eating at America’s Table study (EATS). When compared to 24-h recall estimates, this assessment was found to be accurate for approximate fruit and vegetable intake estimates that can be used to rank individuals [42].

Dietary quality. Dietary quality was assessed via the DASH-Q, a self-report survey measure of adherence to the dietary approaches to stop hypertension (DASH) diet [43]; the DASH diet is associated with reduced risk of cardiovascular disease, mortality, cancer, and type 2 diabetes [44]. The DASH-Q has been validated against measures of diet quality such as the dietary survey tool (DST), which has been validated against 24-h recall estimates [45].

Nutrition knowledge. The weight management nutrition knowledge questionnaire [46] measures knowledge of calorie density, portion size, and food variety. The questionnaire has adequate convergent validity, construct validity, criterion validity, and test-retest reliability. The energy density, portion size, and food variety sub-scales were used.

Food choice. Food choice was assessed with the Multiple Food Test, a validated measure of food choices in online contexts that has adequate psychometric properties and correlates with actual food choices [47]. In the Multiple Choice Food Test, individuals see pictures of four different foods and are asked to choose one they would most prefer to eat right now. There are 24 food pictures in total, comprising four categories ranging from unhealthy to healthy; each category has 6 food pictures. The categories are based on nutrient profiling model (NPM) scores. The nutrient profiling model (NPM), which has been validated against the diet quality index (DQI), is a scoring system based on the UK’s Balance of Good Health guide [48,49]. NPM scores range from −15 (healthy) to +15 (unhealthy), and adds positive points for sugar, saturated fat, and energy, and negative points for fruits, vegetables, nuts, fiber, and protein [48]. Each category in the Multiple Food Test (unhealthy to healthy) is separated from others by at least 3 NPM points. In the computerized test, there are 18 trials, with no practice trials. In each trial, pictures of four foods, one food per category, are randomized but counterbalanced across trials such that they appear in a random order and each food appears an equal number of times across all trials (see [47] for a list of the foods in each category and all images). In a given trial, then, individuals will see four pictures in a random order, each one from each category. Individuals are asked, “Which of the following foods would you choose if these foods were offered to you now?” Scoring in each trial ranges from 1 (unhealthiest food) to 4 (healthiest food). Scores for each chosen food are summed across all 18 trials. Higher scores denote healthier foods.

Weight and energy density. Weekly self-reported weight and food intake for 18 months were extracted from the program database. Weekly and 18 month-average food color (energy density) proportions were calculated from self-reported food data similarly to 4-month data.

### 2.5. Statistical Analyses

Descriptive statistics are expressed in means and standard deviations. For 4-month analyses, one-way ANOVAs were conducted with the weight loss group as the independent variable and energy density proportions as the dependent variable. Variables did not significantly deviate from a normal distribution according to a two-sided one-sample Kolmogorov-Smirnov test. Linear mixed modeling evaluated over-time predictors of energy density proportions, with weekly low-energy-dense food proportions as the dependent variable and weight loss group, time in weeks, baseline BMI, age, gender, and the interaction between time and weight loss group as fixed effects. Random effects were a random slope for time and a random intercept for each participant. For 18-month analyses, one-way ANOVAs were used to compare weight loss groups in energy density proportions, fruit and vegetable intake, dietary quality, nutrition knowledge, and food choice. Two types of regression models were performed [50]. First, each nutritional factor (fruit and vegetable intake, dietary quality, nutrition knowledge, and food choice) was used as an independent variable predicting weight change at 18 months in multiple linear regressions. Second, models were adjusted for covariates of baseline BMI, age, gender, average adherence to calorie budgets, and average low-energy-dense food proportions. This was done to understand influential nutritional factors even when accounting for energy intake, energy density, and baseline characteristics, which all predict weight change [51,52,53]. For this analysis, adherence to calorie budgets was determined by the proportion of days per month that participants reached within 5% of their recommended calorie budget, which is based on their preferred rate of weight change (i.e., 0.5–2 pounds per week), physical activity level, and basal metabolic rate as calculated by the Harris Benedict equation [54]. Calorie budget adherence was calculated only for days in which participants logged all three meals to adjust for bias from missing intake data that can drastically change average calorie amounts. All other variables (i.e., energy density proportions) were not restricted to instances when all meals were logged. Significance tests were two-sided, with an α of 0.05. Analyses were conducted in R (version 3.6.0). 

## 3. Results

### 3.1. 4 Months

The moderate weight loss group was 80% female and 20% male, and the stable weight group was 87% female and 13% male; this proportion varied across weight loss groups (*p* = 0.001), as did height (*p* = 0.007). At baseline, both stable and moderate weight loss groups had a similar initial weight (stable: 102.8 kg, SD = 17.4; weight loss: 101.1 kg, SD = 16.2; *p* = 0.07; Table 1). Baseline BMI differed across groups (stable: 30.7, SD = 4.8; moderate: 30.0, SD = 3.8; *p* < 0.004). At 4 months, the moderate weight loss group lost an average of 9.0 kg (SD = 3.4; 8.9% body weight loss), while the stable weight group lost an average of 0.2 kg (SD = 0.6; 0.2% body weight loss). 

Energy density proportions over 16 weeks were examined by meal (Table 2). The moderate weight loss group consumed a greater proportion of low-energy-dense food than the stable group for all meals (all ps < 0.001). The moderate loss group also consumed lower high-energy-dense food proportions for all meals (all ps < 0.001). Medium-energy-dense food proportions were lower in the moderate group for breakfast but not lunch or dinner (breakfast: *p* < 0.001; lunch: *p* = 0.73; dinner: *p* = 0.44). The moderate loss group also reported ingesting fewer calories at all meals (all ps < 0.001). 

Linear mixed model results indicated that the interaction of time and weight loss group was not significant (B = −0.01, *p* = 0.80; Table 3). There was a main effect of weight loss group, in which weekly low-energy-dense food proportions were higher in the moderate loss group than the stable group (B = 6.67, *p* < 0.001). Age, gender, and baseline BMI were also significant predictors of weekly low-energy-dense food proportions, in which older participants, female participants, and participants with lower baseline BMI ate more low-energy-dense foods (B = 0.13, *p* < 0.001; B = −1.68, *p* < 0.001; B = −0.11, *p* = 0.003). Low-energy-dense food proportions declined over time (B =−0.11, *p* = 0.002). 

### 3.2. 18 Months

Baseline weight, BMI, height, and the proportion of female to male participants did not differ across weight loss groups (high: >10% body weight loss, moderate: 5–10% body weight loss, low: less than 5% body weight loss; Table 1). Race, ethnicity, employment status, education, income, and marital status at 18 months also did not significantly differ across weight loss groups, while age did (high: M = 57.0, moderate: M = 54.7, low: M = 50.3, *p* = 0.03). Baseline BMI was 36.9, 36.0, and 36.5, respectively. Average weight loss was 19.4 kg (SD = 7.9; 19.4% body weight loss) for the high weight loss group, 7.4 kg (SD = 1.7; 7.3% body weight loss) for moderate weight loss, and 1.5 kg (SD = 2.8; 1.5% body weight loss) for low weight loss. 

At 18 months, low-energy-dense food proportions were significantly different across weight loss groups at all meals (all ps < 0.01, Table 2). High-energy-dense food proportions also significantly differed across groups for all meals (all ps < 0.001; Table 2). Medium-energy-dense food proportions significantly differed across weight loss groups for breakfast (*p* < 0.001), but not lunch (*p* = 0.54) or dinner (*p* = 0.68). Post-hoc tests for multiple comparisons using the Bonferroni correction revealed some differences across weight loss groups for breakfast and lunch, but all groups were different from each other for dinner. For breakfast and lunch, only high weight loss individuals had greater low-energy-dense proportions than low weight loss individuals (ps < 0.007). For breakfast, high-energy-dense proportions differed between high and medium groups, as well as high and low groups (ps < 0.001). For lunch, high-energy-dense proportions differed between high and low groups (*p* < 0.001) and medium and low groups (*p* < 0.01). However, for dinner, significant differences emerged across all comparisons (high vs. medium, high vs. low, and medium vs. low) for both low-energy-dense and high-energy-dense proportions ( all ps < 0.001). Calories also significantly differed across all comparisons at all meals (all ps < 0.001).

Eighteen-month fruit and vegetable intake, dietary quality, nutrition knowledge, and food choice significantly differed across weight loss groups (F(2,145) = 3.47, *p* = 0.03); F(2,145) = 4.08, *p* = 0.02; F(2,145) = 9.39, *p* < 0.001; F(2,145) = 6.20, *p* = 0.003; Table 4). Post-hoc tests revealed that there was greater dietary quality and nutrition knowledge among individuals with high compared to low weight loss, as well as medium compared to low weight loss (ps < 0.01). For fruit and vegetable intake and food choice, only the high and low groups were different from each other (*p* < 0.01, *p* < 0.001). 

Regression results are displayed in Table 5. Greater fruit and vegetable intake was associated with decreased weight in the crude model (B = −0.42, *p* = 0.03). This was no longer significant after adjusting for covariates of baseline BMI, age, gender, average adherence to calorie budgets over 18 months (B = −0.28, *p* =.17). Dietary quality was not associated with weight loss in either crude (B = −0.08, *p* = 0.33) or adjusted (B = 0.001, *p* = 0.98) models. Notably, nutrition knowledge was associated with weight loss in both crude (B = −21.59, *p* = 0.003) and adjusted (B = −19.44, *p* = 0.006) models, in which more nutrition knowledge was associated with decreased weight. Similarly, food choice was associated with weight loss in both crude (B = −5.48, *p* < 0.001) and adjusted (B = −5.49, *p* = 0.002) models. Healthier food choice was associated with decreased weight.

## 4. Discussion

To our knowledge, this is the first study examining nutritional factors, such as energy density, fruit and vegetable intake, diet quality, and nutrition knowledge, on a self-managed commercial weight loss program. Using a program with a food color categorization system based on energy density, we examined how daily energy density food proportions related to weight lost at 4 months and 18 months in a retrospective analysis, and how nutritional factors related to weight loss at 18 months in a cross-sectional survey. Compared to participants who lost less weight, participants with greater weight loss at 18 months (>10%, 5–10%) and 4 months (≥5%) ate greater proportions of low-energy-dense foods and smaller proportions of high-energy-dense foods. At 18 months, the differences across groups were most pronounced for dinner foods. These results corroborate previous studies showing that individuals in RCT and self-managed contexts without a program had greater weight loss when eating low-energy-dense foods [20,22,55]. In addition, participants at 18 months with greater weight loss (5–10%, 10%) had significantly higher self-reported fruit and vegetable intake, dietary quality, nutrition knowledge, and healthier food choice. This aligns with qualitative evidence that individuals with greater long-term weight loss have better dietary quality and nutrition knowledge than individuals who have not maintained weight loss [14,56,57]. Despite its limitations, this study is an important first step in ascertaining whether similar relationships are found in more self-managed environments, particularly long-term. 

We also found that of factors studied, only nutrition knowledge and food choice were associated with weight change in both crude and adjusted models. This is consistent with other studies showing that food choices at ad libitum buffets predict weight loss and that weight loss is most associated with increased intake (i.e., choice) of healthy foods and reduced consumption of foods such as dessert, red meat, and cheese in free-living conditions [58,59,60]. In the Multiple Food Test used in this study, among choices such as meat, cheese, dessert, fruit, vegetables, and more, individuals who chose the healthiest foods had greater weight loss. For the first time, our results highlight the potential importance in weight loss of initial inclinations towards food based on depictions as in the Multiple Food Test but not actual consumption of test foods. Because of the small sample size involved in this study, future results should clarify whether is associated with long-term weight loss in other samples. Our results on nutrition knowledge also build on previous studies showing that for individuals in free living conditions and clinical trials, nutrition knowledge is associated with greater weight loss and weight control behaviors [9,61]. Our results also contribute to a small body of work showing that energy density predicted weight change the most out of factors such as baseline BMI or caloric intake [22,62]. In this study, dietary quality was not a significant predictor while nutrition knowledge and food choice were, which highlights the need for research that investigates the effects of more nutritional factors when exploring effects of energy density.

Unexpectedly, dietary quality and fruit and vegetable intake were not significantly associated with weight loss, though they differed by weight loss in expected ways. Dietary quality, assessed as adherence to the DASH diet, was not significantly associated with weight loss in either model. Previous studies indicate associations between dietary quality, whether measured as DASH diet adherence or not, and weight loss [48,63,64,65], though a meta-analysis suggests fruit and vegetable intake is not associated with weight loss [66]. Our results demonstrate for the first time to our knowledge that fruit and vegetable intake is associated with weight loss in an individual model but not in an adjusted model when accounting for baseline characteristics, energy density, and average adherence to calorie budgets. Future research should investigate whether these other characteristics influence weight loss more than fruit and vegetable intake on its own or adhering to a diet such as DASH. This is particularly the case since some fruit and vegetables included in the DASH diet (i.e., avocados, figs) may add calories but not enough volume to be helpful for weight loss. However, our results could be due to lack of variance from the short survey measures used, even though they have been validated against longer dietary assessments, or from the fact that all weight groups had moderate DASH-Q scores compared to previous studies [67,68].

In the linear mixed model at 4 months and regression models at 18 months, we accounted for age and gender, since each can be associated with weight loss [5,69]. However, there could be other variables that we did not capture, such as socioeconomic factors, that may be associated with 18-month weight loss. In a previous study, socioeconomic factors such as income and education did not emerge in a stepwise regression as important factors associated with weight loss at 4 months on Noom [6]. However, one study found that socioeconomic factors are associated with maintenance of long-term weight loss [70]. In another study, individuals with obesity who used a commercial program and maintained weight loss had higher income and were more likely to be employed and college educated than individuals with obesity who were weight stable and did not use a commercial program [71]. Future research on weight loss in a commercial program should account for as many factors as possible, including but not limited to socioeconomic factors.

This was an observational retrospective investigation seeking to provide knowledge based on a context as close as possible to how individuals use a digital commercial program in the real world (i.e., not within the context of a clinical trial or prospective study). This is a first step towards informing improved nutritional intake and knowledge on these programs. However, this approach has limitations. For example, there was no control group, which would illuminate how the program improves nutritional factors compared to usual care. This limits generalizability to those who pay for a commercial program. Moreover, intent-to-treat analyses were not used, which means that results are limited to those who responded to the survey and met inclusion criteria, and may not generalize well to those who would not have met this criteria. In addition, we could not measure how self-reported nutritional factors changed from baseline to 4 months and 18 months. In using a cross-sectional observational cohort design, we could not assess how any factors predict subsequent weight loss. Nutritional factors were assessed via short self-reported assessments, which could mean that fruit and vegetable intake and dietary quality measures lack precision and fail to pick up on variation that would be related to weight loss. Though the direction of results across weight loss groups indicates that they were sensitive enough to pick up differences, future research should use longer assessments to measure dietary intake and quality. Additionally, weight was self-reported rather than objectively measured. Energy density proportions were also based on self-reported food intake during the program. Self-reported food intake is often subject to underreporting [72]. Future studies should examine the extent of underreporting across weight loss groups in detail and particularly take into account the source of inaccuracy (e.g., deliberate and unintentional underreporting, as opposed to changed and motivated eating behavior after learning from a program; [72]).

For 4-month analyses, only participants in one of two weight loss groups (5% or more, 0 ± 1%) were included. For 18-month analyses, only participants who were from this original sample and who were still on the program at 18 months (25%), who responded to the survey (30% response rate), and who self-reported weight between weeks 68–74 (60% of the remaining participants) were included. This likely represents a minority of individuals on commercial programs, limiting generalizability. This also means the sample could be biased in that these participants were likely more motivated than users who do not stay on the program for 18 months. Retention rates in weight loss programs tend to vary considerably, and can range from 20% to over 90% [73,74,75]. Retention through the study was low but comparable to the low range for other weight loss interventions [73,74,75]. This could be due to a few reasons. First, unlike interventions that have a set time period from the start, participants could choose how long they wanted to use the program. In addition, retention can drop drastically over time, particularly when considering long-term weight loss; one study found that retention in a commercial weight loss program was more than 70% at 4 weeks but 6.6% at one year [76]. The survey response rate (30%) is also comparable to those found in the survey methodology literature for online surveys (e.g., 35%; [77]). Of the remaining participants, 60% were still actively self-reporting weight, which is comparable to rates of self-monitoring in other studies (e.g., 65%, [78]). Future studies should compare these usage and retention factors between those who continue in this type of commercial program and those who do not. 

## 5. Conclusions

This study is the first to our knowledge exploring long-term nutritional factors in a self-managed commercial program, finding that nutritional factors were related to weight loss by 18 months. The results suggest that individuals with higher amounts of weight loss can maintain lower-energy-dense and higher quality food intake, greater fruit and vegetable intake, and better food choices even 18 months after starting the program. Our results provide a preliminary step towards better understanding factors in real-world use of a digital commercial program but should be interpreted with caution given the retrospective design and the small number of participants who remained in the study at 18 months. Our results also suggest for the first time that the preference for images of healthy vs. unhealthy foods was associated with long-term weight loss. This could be used by interventions to identify individuals who likely have more or less sustained weight loss; however, future research should provide further evidence for this notion by using randomized controlled trials with pre- and post- measurements. At both 4 months and 18 months, individuals who lost more weight on a digital commercial program reported consuming a higher proportion of low-energy-dense foods than individuals who lost less weight. Finally, our results provide the first evidence that nutrition knowledge and food choice are key factors associated with long-term weight loss in this type of program. Even though it is known that nutrition knowledge and energy density are generally associated with weight loss, this knowledge has only been derived from non-commercial settings. Our results provide rare data on relationships between nutritional factors and weight loss on a digital commercial program. Taken together, these findings reveal how nutritional factors after long-term use of a self-managed commercial weight loss program relate to weight loss and also can inform efforts towards more optimal nutritional outcomes on this type of program. 

## Figures and Tables

**Table 1:** Baseline characteristics of 4-month and 18-month participants.

**4 Months**				
**Characteristic**		**Moderate Weight Loss (≥5%) (*n* = 2887)**	**Stable Weight (0 ± 1%) (*n* = 374)**	***p* Value**
**Gender, *n* (%)**				
Male		585 (20.3)	49 (13.1)	0.001
Female		2302 (79.7)	325 (86.9)	
Age (years), mean (SD)		51.0 (12.4)	49.7 (12.5)	0.07
Initial weight (kg), mean (SD)		101.1 (16.2)	102.8 (17.4)	0.07
Height (inches), mean (SD)		66.3 (3.6)	65.8 (3.3)	0.007
Baseline BMI (kg/m^2^), mean (SD)		30.0 (4.3)	30.7 (4.8)	0.004
Average weight loss (kg) at 4 months, mean (SD)		9.0 (3.4)	0.2 (0.6)	<0.001
Average weight loss (%) at 4 months, % (SD)		8.9% (3.0%)	0.2% (0.6%)	<0.001
**18 months**				
**Characteristic**	**High weight loss (>10%) (*n* = 71)**	**Moderate weight loss (5–10%) (*n* = 35)**	**Low weight loss (less than 5%) (*n* = 42)**	***p* value**
**Gender, *n* (%)**				0.93
Male	13 (18%)	7 (20%)	7 (17%)	
Female	58 (82%)	28 (80%)	35 (83%)	
Age (years), mean (SD)	57.0 (10.9)	54.7 (15.5)	50.3 (12.3)	0.03
Initial weight (kg), mean (SD)	103.1 (15.8)	102.3 (14.0)	100.6 (14.0)	0.68
Height (inches), mean (SD)	66.5 (3.1)	66.9 (3.6)	65.9 (2.9)	0.37
Baseline BMI (kg/m^2^), mean (SD)	36.9 (5.9)	36.0 (4.1)	36.5 (4.6)	0.68
Average weight loss (kg) at 18 months, mean (SD)	19.4 (7.9)	7.4 (1.7)	1.5 (2.8)	<0.001
Average weight loss (%) at 18 months	19% (7.1%)	7.3% (1.3%)	1.5% (2.7%)	<0.001

Note: There were no missing values since only individuals with complete baseline data and at least one meal or weigh-in per week were included.

**Table 2:** Energy density proportions at 4 months and 18 months.

**4 Months**				
**Characteristic**		**Moderate Weight Loss (*n* = 2887)**	**Stable Weight (*n* = 374)**	***p* Value**
		**Mean (SD)**	**Mean (SD)**	
**Breakfast**				
Calories per meal		255.9 (103.4)	288.5 (127.1)	<0.001
Low-energy-dense proportion		0.35 (0.26)	0.25 (0.24)	<0.001
Medium-energy-dense proportion		0.33 (0.23)	0.35 (0.25)	<0.001
High-energy-dense proportion		0.28 (0.23)	0.36 (0.25)	<0.001
**Lunch**				
Calories per meal		373.0 (139.8)	413.2 (162.9)	<0.001
Low-energy-dense proportion		0.23 (0.17)	0.16 (0.15)	<0.001
Medium-energy-dense proportion		0.43 (0.2)	0.43 (0.22)	0.73
High-energy-dense proportion		0.30 (0.19)	0.38 (0.22)	<0.001
**Dinner**				
Calories per meal		492.6 (179.1)	508.7 (239.6)	<0.001
Low-energy-dense proportion		0.19 (0.14)	0.14 (0.14)	<0.001
Medium-energy-dense proportion		0.45 (0.18)	0.45 (0.21)	0.44
High-energy-dense proportion		0.32 (0.19)	0.38 (0.22)	<0.001
**18 months**				
	**High weight loss (>10%) (*n* = 71)**	**Moderate weight loss (5–10%) (*n* = 35)**	**Low weight loss (less than 5%) (*n* = 42)**	***p* value**
**Breakfast**				
Calories per month	15,636.6 (9245.4) ^a^	14,026.4 (8200.4) ^a^	11,629.2 (7793.8) ^a^	<0.001
Low-energy-dense proportion	0.37 (0.26) ^a^	0.37 (0.24) ^b^	0.34 (0.25) ^ab^	0.02
Medium-energy-dense proportion	0.41(0.24) ^a^	0.36 (0.21) ^ab^	0.4 (0.24) ^b^	<0.001
High-energy-dense proportion	0.21 (0.2) ^ab^	0.27 (0.21) ^b^	0.26 (0.19) ^a^	<0.001
**Lunch**				
Calories per month	19,652.4 (9753.7) ^a^	17,760.5 (9264.9) ^a^	14,814.0 (9705.4) ^a^	<0.001
Low-energy-dense proportion	0.29 (0.16) ^a^	0.27 (0.16)	0.25 (0.18) ^a^	<0.001
Medium-energy-dense proportion	0.37 (0.16)	0.38 (0.16)	0.37 (0.18)	0.54
High-energy-dense proportion	0.34 (0.18) ^a^	0.35 (0.19) ^ab^	0.37 (0.21) ^ab^	0.002
**Dinner**				
Calories per month	27,922.2 (14495.4) ^a^	23,261.7 (14505.0) ^a^	20,145.6 (14120.7) ^a^	<0.001
Low-energy-dense proportion	0.22 (0.15) ^a^	0.20 (0.14) ^a^	0.16 (0.13) ^a^	<0.001
Medium-energy-dense proportion	0.42 (0.15)	0.42 (0.16)	0.41 (0.18)	0.68
High-energy-dense proportion	0.36 (0.17) ^a^	0.39 (0.18) ^a^	0.43 (0.21) ^a^	<0.001

Note. Shared superscripts denote significant differences at *p* < 0.05 in Bonferroni-corrected post-hoc analyses.

**Table 3:** Linear mixed models predicting weekly low-energy-dense proportions for 4-month participants.

	Low-Energy-Dense Proportion			
Characteristic	Coefficient	95% CI	SE	*p* Value
**Group**				
Stable	-	-	-	-
Weight loss group	6.67	5.64, 7.70	0.53	<0.001
Time	−0.11	−0.18, 0.04	0.04	0.002
Age	0.13	0.11, 0.16	0.01	<0.001
**Gender**				
Female	-	-	-	-
Male	−1.68	−2.45, −0.91	0.39	<0.001
Baseline BMI	−0.11	−0.18, −0.04	0.03	0.003
**Time** × **Group**				
Time × Stable	-	-	-	-
Time × Weight loss group	−0.01	−0.08, 0.06	0.04	0.80

**Table 4:** Differences in nutritional factors among weight loss groups at 18 months.

	High Weight Loss (>10%) *n* = 71	Moderate Weight Loss (5–10%) *n* = 35	Low Weight Loss (Less than 5%) *n* = 42	*p* Value
Dietary quality (Range: 0 to 77)	41.8 (10.5) ^a^	42.5 (9.4) ^b^	36.9 (9.1) ^ab^	0.02
Fruit/vegetable intake (Range: 0 to 30)	7.2 (4.7) ^a^	6.3 (2.3)	5.1 (3.8) ^a^	0.03
Nutrition knowledge (% correct)	82% (9%) ^a^	83% (11%) ^b^	74% (12%) ^ab^	<0.001
Food choice (range: 0 to 4)	3.0 (0.4) ^a^	2.9 (0.5)	2.7 (0.5) ^a^	0.003

Note. Shared superscripts denote significant differences at *p* < 0.05 in Bonferroni-corrected post-hoc analyses. For fruit/vegetable intake, scores represent the frequency of vegetable and fruit items per week as a function of the serving size. An example frequency range is never → more than 5 times a day, and an example serving size range is less than ¾ cup → more than 2 cups.

**Table 5:** Crude and adjusted regression models predicting weight change at 18 months.

Predictor	Model	Coefficient (95% CI)	Std. Error	T Value	*p* Value
Dietary quality	crude	−0.08 (−0.24, 0.08)	0.08	−0.96	0.33
	adjusted	0.00 (−0.17, 0.17)	0.09	0.02	0.98
Fruit/veggie intake	crude	−0.42 (−0.81, −0.04)	0.19	−2.17	0.03
	adjusted	−0.28 (−0.68, 0.12)	0.2	−1.38	0.17
Nutrition knowledge	crude	−21.59 (−35.58, −7.61)	7.08	−3.05	0.003
	adjusted	−19.44 (33.19, −5.69)	6.96	−2.79	0.006
Food choice	crude	−5.48 (−8.67, −2.28)	1.62	−3.38	0.001
	adjusted	−5.49 (−8.87, −2.11)	1.71	−3.21	0.002

Note. In crude models, each predictor was an individual independent variable, with weight change at 18 months as the dependent variable. In adjusted models, covariates were baseline BMI, age, gender, average calorie budget adherence over 18 months, and average low-energy-dense food proportions over 18 months.

## Data Availability

Restrictions apply to the availability of these data. Data was obtained from Noom and are available by request from the corresponding author with the permission of Noom.
